# Renal quality outcomes framework and eGFR: impact on secondary care

**DOI:** 10.1186/1753-6561-6-S4-O44

**Published:** 2012-07-09

**Authors:** LA Phillips, KL Donovan, AO Phillips

**Affiliations:** 1 Institute of Nephrology, Cardiff University School of Medicine, Heath Park, Cardiff, UK

## Introduction

The recognised benefits of early detection and management of chronic kidney disease (CKD), led to the introduction of estimated glomerular filtration rate (eGFR) reporting and the incorporation of CKD into the revised Quality Outcomes Framework (QOF) of the General Medical Servcies (GMS) contract in the UK . Our aim was to characterize the effect of these changes on referral numbers and appropriateness to a nephrology service, and to assess the impact of a new patient care pathway. In addition, we reviewed the Welsh 2006-8 CKD QOF data, to explore its relation to population demographics and relevant QOF data.

## Methods

Analysis of referrals was based on data from one NHS Trust covering five Local Health Boards (LHBs). In addition, we compared data from all Welsh CKD QOF returns (2006-8) with biochemistry data, QOF data for related chronic diseases, and published epidemiology. Correlations between LHB prevalence of CKD, CHD, diabetes, hypertension and social deprivation were analysed using linear regression. A p value of <0.05 was considered significant.

## Results

Introduction of eGFR reporting and CKD QOF domains led to a 61% increase in referrals, and an increase in patient age at referral (63.0±18.1 to 69.1±18.5.) Screening of referrals demonstrated 36% to be inappropriate or inadequate in terms of clinical information supplied, but this fell following the introduction of the patient care pathway. The QOF prevalence of CKD in 2007 (2.4%) and 2008 (2.9%) was lower than that derived from local laboratory data (6 , 9%) and published studies (6 , 8%) and did not correlate with related chronic medical conditions. The prevalence of CKD varied markedly between Local Health Board (LHB) regions (1.7%–3.7% in 2007, 1.8%–4.2% in 2008).

## Conclusions

Our data demonstrates the increase in workload in nephrology outpatients since the introduction of eGFR reporting and CKD QOF domains. Analysis of the QOF data show marked inconsistencies, particularly between reported CKD prevalence and established risk factors, thus raising concerns about the reliability of the data and the clinical care it purports to underpin.

